# Effects of age on pregnancy outcomes in patients with simple tubal factor infertility receiving frozen-thawed embryo transfer

**DOI:** 10.1038/s41598-020-75124-3

**Published:** 2020-10-22

**Authors:** Yi-Fei Sun, Jie Zhang, Yue-Ming Xu, Zhuo-Ye Luo, Yong Sun, Gui-Min Hao, Bu-Lang Gao

**Affiliations:** Department of Reproductive Medicine, The Second Hospital of Hebei Medical University, 215 West Heping Road, Shijiazhuang, 050011 Hebei China

**Keywords:** Biological techniques, Biotechnology, Developmental biology, Diseases, Health care, Medical research

## Abstract

This study was to retrospectively analyze the effect of the age of embryos transfer and oocyte retrieval on the clinical pregnancy outcome in patients with simple tubal factor infertility (TFI) who received frozen-thawed embryo transfer. Patients (n = 3619) with simple TFI who underwent in vitro fertilization (IVF)/ intracytoplasmic sperm injection (ICSI) frozen-thawed embryo transfer at our hospital were enrolled. Univariate logistic regression analysis, categorical multivariate logistic regression analysis, curve fitting and threshold effect analysis were performed. Age of embryo transfer was a significant (*P* < 0.05) independent risk factor affecting the clinical pregnancy, live birth, and miscarriage rates. The Clinical pregnancy outcome declined significantly after the age of 34 years. After limiting the female oocyte retrieval age to ≤ 34 years, no significant change was detected in the clinical pregnancy, live birth, or miscarriage rate with increase of transplantation age. In conclusion, in patients with simple TFI undergoing IVF/ICSI frozen-thawed embryo transfer, age is a significant independent risk factor affecting the clinical pregnancy, live birth, and miscarriage rate. Aging of oocytes has a greater impact on the clinical pregnancy in women with simple TFI than the aging of the body. Patients with TFI can freeze embryos in advance to preserve fertility.

## Introduction

As a place where sperm and oocytes are combined, the fallopian tube has the function of picking up eggs and transporting early embryos. Tubal infertility refers to a type of infertility in which women cannot be fertile due to blockage, adhesion, and patency of the fallopian tubes. Many factors can cause tubal infertility, including acute or chronic salpingitis, tubal site pregnancy, tubal site surgery history, appendicitis surgery history^1^, induced abortion history^2^, and endometriosis^3^. All these factors can result in tubal adhesions, obstruction, dysfunction^4^, and subsequently tubal infertility which is one of the most common causes of women infertility. About 12% of infertile couples suffer from this condition^5^. For patients with simple tubal factor infertility (TFI) due to blocked access to sperm and oocytes, assisted reproductive technology (ART) such as in vitro fertilization (IVF)/ intracytoplasmic sperm injection (ICSI) has become a non-negligible option.

Age is one of the non-ignorable risk factors to affect the clinical pregnancy outcome in patients with simple TFI. After studying 731 infertile patients with involuntary infertility whose partners had sperm abnormalities, Dunphy et al.^6^ found that female age was positively correlated with the incidence and severity of tubal occlusive diseases. Balasch et al^7^ also found that for women over 35 years of age, the probability of infertility due to tubal factors increased, the miscarriage rate also increased, but the pregnancy rate decreased. Schippert et al^8^ recommended surgery to treat fallopian tube diseases for patients under 36 years old in good condition or patients under 40 years old undergoing fallopian tube recanalization after birth control surgery. ART should be selected for patients over 36 years old who have not undergone fallopian tube recanalization after birth control surgery. However, female fertility gradually declines with aging, and begins to decline significantly at about 32 years of age, with the declining sped up greatly after 37 years old^9^. How to save female fertility and choose the best treatment at the right age so as to obtain the best clinical outcome is worthy of further investigation. To do this, the effect of age of embryo transfer and oocyte retrieval should be clearly understood on the clinical pregnancy outcome in patients with simple TFI who are to receive frozen-thawed embryo transfer. This study was consequently performed to analyze the effect of age on clinical pregnancy in this kind of patients.

## Results

### General conditions and pregnancy outcomes

The infertility duration, number of pregnancies, body mass index (BMI), basal follicle-forming hormone (bFSH), estradiol (E2) on the trigger day, number of oocytes retrieved, and the level of two embryos transferred were significantly higher in the younger (≤ 32 years) than in the older group (> 32 years), respectively (Table 1). The levels of basic luteinizing hormone (bLH), bLH/ (basal follicle-forming hormone) FSH, basic anti-mullerian hormone (bAMH), progesterone (P) on the trigger day, and three embryos transferred were significantly (*P* < 0.05) lower in the younger than in the older group, respectively. The clinical pregnancy rate and live birth rate were significantly (*P* < 0.05) higher in the younger than in the older group, whereas the miscarriage rate was significantly (*P* < 0.05) lower in the younger than in the older group. The number of oocytes retrieved was significantly (*P* < 0.05) greater in the younger than in the older group. No significant differences were detected in the levels of basic estradiol (bE2), endometrial thickness on the trigger day, luteinizing hormone (LH) on trigger day, ectopic pregnancy rate, durationg of embryo freezing, and number of one embryo transferred between the two groups (Table 1).

**Table 1:** Demography and clinical pregnancy outcomes of the patients.

Group	> 32	≤ 32	*P*
N	914	2705	
Infertility duration	5.09 ± 3.72	3.33 ± 2.04	< 0.05
Number of pregnancies (median + range)	1.00 ± 2.00	0.00 ± 1.00	< 0.05
BMI (kg/m^2^)	23.54 ± 3.13	23.19 ± 3.57	< 0.05
bFSH (mIU/ml)	7.48 ± 2.68	6.90 ± 1.97	< 0.05
bE_2_ (pg/ml)	45.33 ± 40.80	46.79 ± 43.29	> 0.05
bLH (mIU/ml)	5.04 ± 3.22	5.95 ± 4.56	< 0.05
bLH/FSH	0.89 ± 3.08	0.98 ± 1.87	< 0.05
bAMH (ng/ml)	3.81 ± 3.29	5.53 ± 3.72	< 0.05
Endometrial thickness on trigger day (mm)	9.42 ± 1.52	9.54 ± 1.57	> 0.05
E_2_ on trigger day (pg/ml)	400.64 ± 421.83	355.45 ± 342.79	< 0.05
LH on trigger day (mIU/ml)	21.66 ± 24.86	20.54 ± 17.66	> 0.05
P on trigger day (ng/ml)	0.54 ± 0.41	0.60 ± 0.41	< 0.05
**Number of embryos transferred**			
1	6.67% (61/914)	5.58% (151/2705)	> 0.05
2	77.02% (704/914)	81.85% (2214/2705)	< 0.05
3	16.30% (149/914)	12.57% (340/2705)	< 0.05
Clinical pregnancy rate	67.40% (616/914)	74.90% (2026/2705)	< 0.05
Live birth rate	51.64% (472/914)	60.19% (1628/2705)	< 0.05
Miscarriage rate	20.94% (129/616)	16.44% (333/2026)	< 0.05
Ectopic pregnancy rate	2.44% (15/616)	3.21% (65/2026)	> 0.05
Number of oocytes retrieved	14.01 ± 7.54	17.60 ± 7.97	< 0.05
Years of embryo freezing (y)	0.00 ± 1.00	0.00 ± 1.00	> 0.05

BMI, Body Mass Index; bFSH, basal follicle-stimulating hormone; bE2 , baseline estradiol; bLH, baseline luteinizing hormone; bLH/FSH, baseline luteinizing hormone / follicle-stimulating hormone; bAMH, baseline anti-Mullerian hormone; E_2_, estradiol on trigger day; LH, luteinizing hormone; P on trigger day, progesterone on trigger day.

### Logistics regression analysis

**Figure 1:**
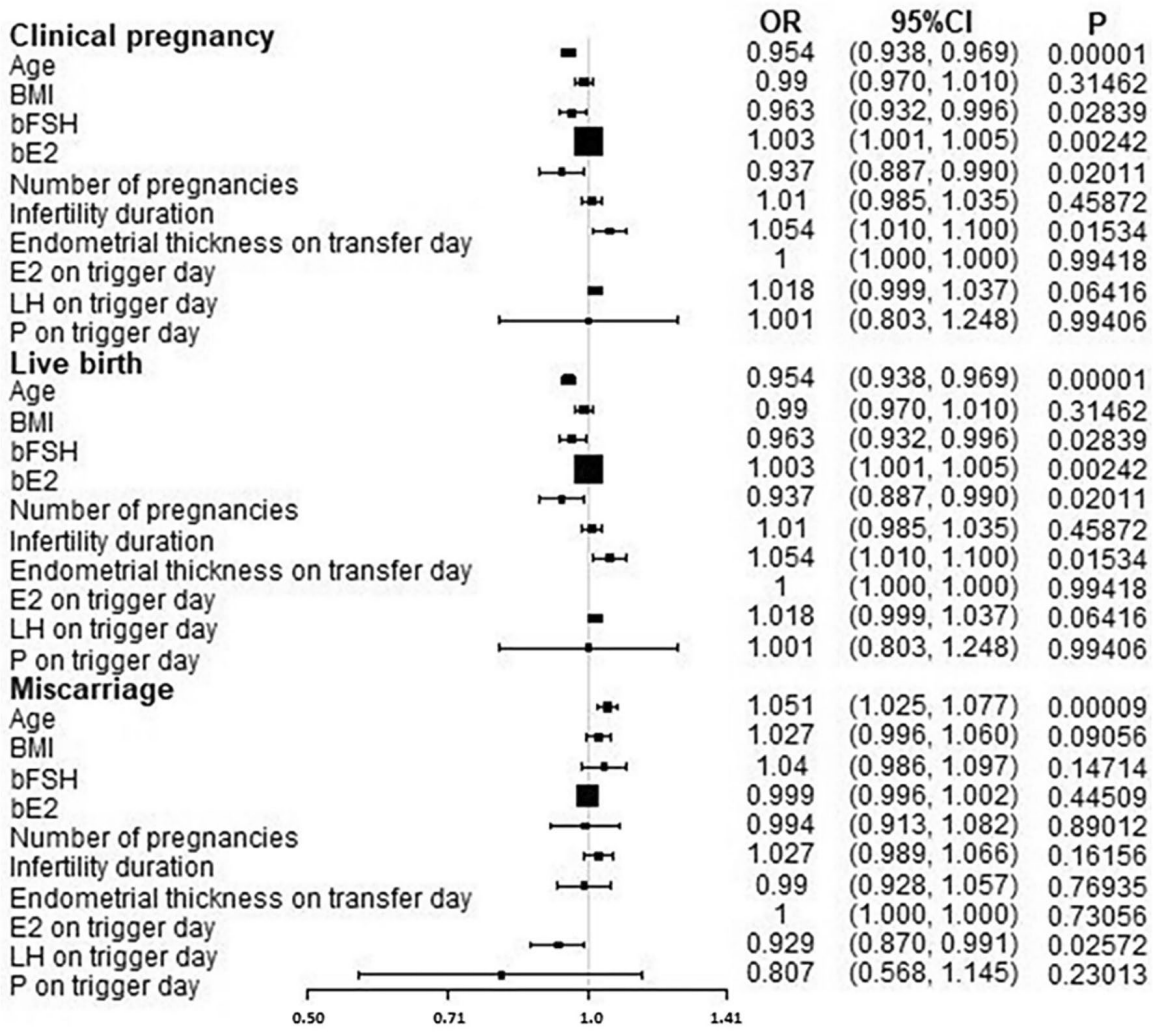
Univariate logistics regression analysis for predicting clinical pregnancy outcome. OR, odds ratio; BMI, Body Mass Index; bFSH, basal follicle-stimulating hormone; bE2, baseline estradiol, bLH, baseline luteinizing hormone; E2, estradiol; LH, luteinizing hormone; P on trigger day, progesterone on trigger day.

Univariate logistics regression analysis demonstrated that age, bE2, infertility duration, number of pregnancies, and endometrial thickness on the trigger day had a significant (*P* < 0.05) effect on the clinical pregnancy rate. Age, bFSH, bE2, number of pregnancies, and endometrial thickness on the trigger day had a significant (*P* < 0.05) effect on the live birth rate, while age, number of oocytes retrieved, and LH on the trigger day had a significant (*P* < 0.05) effect on the abortion rate (Table 2 and Fig. 1).

**Table 2:** Multivariate logistic regression analysis after adjusting confounding factors.

	Non-adjusted, OR (95% CI)	*P*	Adjusted, OR (95% CI)	*P*
**Clinical pregnancy**				
Age (y)	0.955 (0.938, 0.972)	< 0.05	0.940 (0.919, 0.961)	< 0.05
bE_2_ (pg/ml)	1.004 (1.002, 1.006)	< 0.05	1.004 (1.002, 1.007)	< 0.05
Infertility duration	1.031 (1.002, 1.061)	< 0.05	1.058 (1.023, 1.095)	< 0.05
Number of pregnancies	0.915 (0.862, 0.971)	< 0.05	1.014 (0.941, 1.093)	> 0.05
Endometrial thickness on trigger day (mm)	1.049 (1.000, 1.100)	< 0.05	1.045 (0.991, 1.102)	> 0.05
bFSH (mIU/ml)	0.974 (0.940,1.009)	> 0.05		
bAMH (ng/ml)	1.003 (0.892,1.127)	> 0.05		
Number of oocytes retrieved	1.004 (0.988,1.021)	> 0.05		
**Live birth**				
Age (y)	0.954 (0.938, 0.969)	< 0.05	0.961 (0.943, 0.979)	< 0.05
bFSH (mIU/ml)	0.963 (0.932, 0.996)	< 0.05	0.975 (0.942, 1.009)	> 0.05
bE_2_ (pg/ml)	1.003 (1.001, 1.005)	< 0.05	1.003 (1.001, 1.004)	< 0.05
Number of pregnancies	0.937 (0.887, 0.990)	< 0.05	0.990 (0.928, 1.057)	> 0.05
Endometrial thickness on trigger day (mm)	1.054 (1.010, 1.100)	< 0.05	1.056 (1.007, 1.108)	< 0.05
bAMH (ng/ml)	1.002 (0.970,1.036)	> 0.05		
Number of oocytes retrieved	1.007 (0.995,1.019)	> 0.05		
**Miscarriage**				
Age (y)	1.051 (1.025, 1.077)	< 0.05	1.133 (1.060, 1.212)	< 0.05
Number of oocytes retrieved	0.972 (0.958,0.987)	< 0.05	0.984 (0.942,1.028)	> 0.05
LH on trigger day (mIU/ml)	0.929 (0.870, 0.991)	< 0.05	1.001 (0.995, 1.007)	> 0.05
bFSH (mIU/ml)	1.040 (0.986,1.097)	> 0.05		
bAMH (ng/ml)	0.972 (0.873,1.083)	> 0.05		
BMI (kg/m^2^)	1.027 (0.996, 1.060)	> 0.05		

OR, odds ratio; CI, confidence interval; BMI, Body Mass Index; bFSH, basal follicle-stimulating hormone; bAMH, baseline anti-Mullerian hormone; bE_2_, baseline estradiol; LH, luteinizing hormone.

After adjusting confounding factors such as BMI, bE2, infertility duration, number of pregnancies, LH on the trigger day, number of oocytes retrieved, and endometrial thickness on trigger day, multivariate logistic regression analysis showed that age was an independent risk to significantly (*P* < 0.05) affect the clinical pregnancy rate (OR = 0.940, 95% CI: 0.919, 0.961), live birth rate (OR = 0.961,95% CI: 0.943, 0.979), and miscarriage rate (OR = 1.133, 95% CI:1.060, 1.212) (Table 2). In addition, bE2 (OR = 1.004, 95%CI: 1.002,1.007) and infertility duration (OR = 1.058, 95% CI: 1.023,1.095) were two independent risk factors significantly (*P* < 0.05) affecting clinical pregnancy rates, and bFSH (OR = 0.975, 95% CI: 0.942,1.009) was an independent risk factor to significantly affect the live birth rate.

### Smooth curve fitting

**Figure 2:**
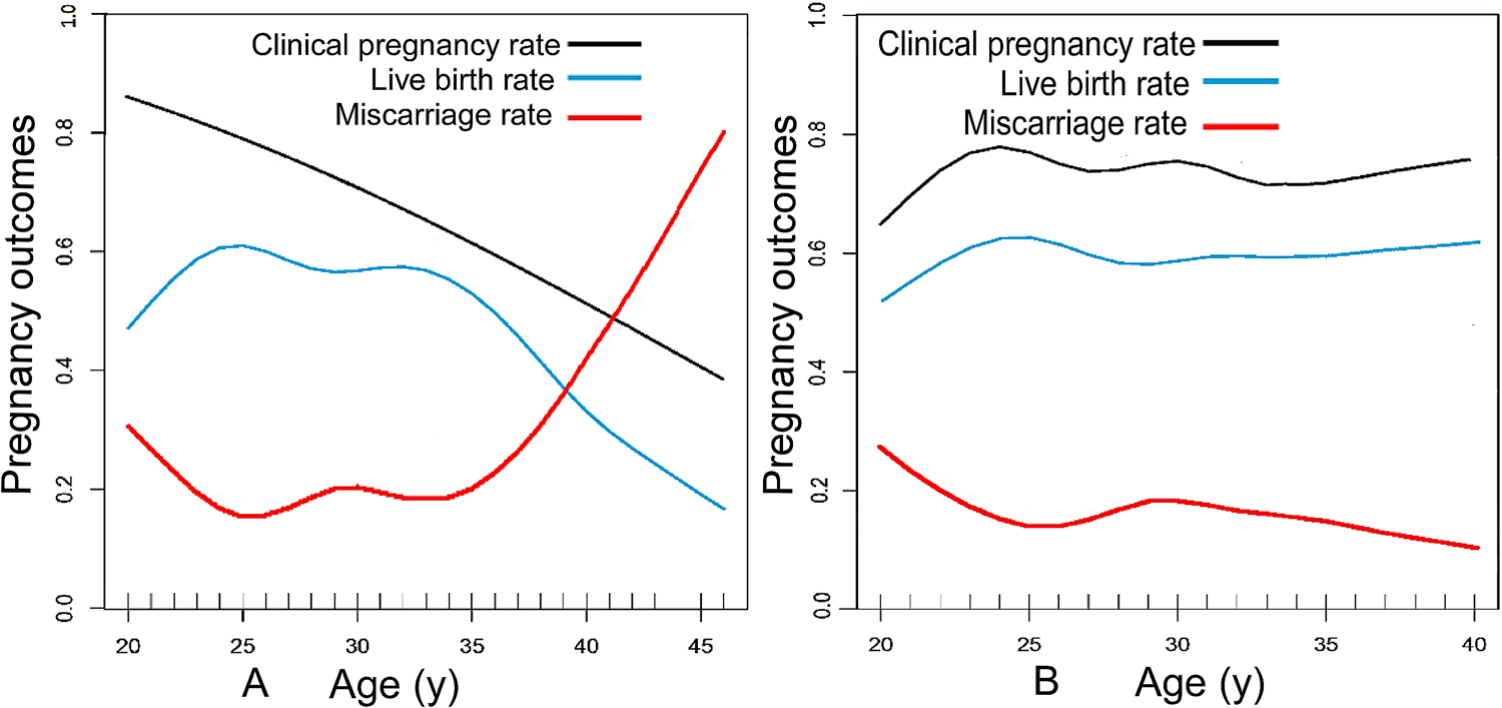
A. Curve fitting diagram of female age and clinical pregnancy outcome. B. Curve fitting diagram of female age and clinical pregnancy outcome in women ≤ 34 years old when taking oocytes.

Smooth curve fitting analysis revealed that the clinical pregnancy rate decreased approximately linearly with increase of female transplantation age when the age of female oocyte retrieval was not restricted. The live birth rate fluctuated at a relatively high level prior to the age of 34 years but fell rapidly after 34 years of age. The miscarriage rate fluctuated at a low level before the age of 35 years but increased linearly after about 35 years old (Fig. 2A). If the oocyte-retrieval age was limited to no more than 34 years, the clinical pregnancy rate and live birth rate fluctuated at a higher level without a significant downward trend as the age of embryos transfer increased, whereas the abortion rate remained at a low level without a significant upward trend (Fig. 2B).

### Threshold effect analysis

After adjusting confounding factors including bE2, infertility duration, and endometrial thickness on the trigger day, without limiting the age of oocyte retrieval, the threshold effect analysis showed that the clinical pregnancy rate decreased significantly (*P* < 0.05) by 6.1% (95%CI: 0.920, 0.957) for every one year increase in the age of female embryo transfer. With every one year increase in age, the live birth rate increased significantly (*P* < 0.05) by 36.2% (95% CI: 1.044, 1.776) for women ≤ 24 years of age, dropped significantly (*P* < 0.05) by 3.2% (95% CI: 0.937, 1.000) for women between 24 and 34 years of age, and further decreased significantly (*P* < 0.05) by 14.5% (95% CI: 0.801, 0.912) for women > 34 years of age (Table 3). The miscarriage rate did not change significantly with increase of age for women < 35 years of age, but increased significantly (*P* < 0.05) by 23% (95% CI: 1.124, 1.347) with every one-year increase in age for women > 35 years of age. After limiting the female oocyte retrieval age to ≤ 34 years, no significant change was detected in the clinical pregnancy, live birth, or miscarriage rate with increase of transplantation age (Table 4).

**Table 3:** Threshold effect analysis of relationship of female age with clinical outcomes.

	OR	95%CI	*P*
**Clinical pregnancy rate**			
Age	0.939	(0.920, 0.957)	< 0.05
**Live birth rate**			
Age< 24	1.362	(1.044, 1.776)	< 0.05
24 ≤ age ≤ 34	0.968	(0.937, 1.000)	< 0.05
Age> 34	0.855	(0.801, 0.912)	< 0.05
**Miscarriage rate**			
Age< 24	0.731	(0.522, 1.024)	> 0.05
24 ≤ age ≤ 35	1.029	(0.989, 1.070)	> 0.05
Age> 35	1.230	(1.124, 1.347)	< 0.05

OR, odds ratio; CI, confidence interval.

**Table 4:** Analysis of relationship of female age with clinical outcome in women ≤ 34 years old when taking oocytes.

	OR	95% CI	P
Clinical pregnancy rate and Age	0.983	(0.959, 1.008)	> 0.05
Live birth rate and Age	0.993	(0.971, 1.015)	> 0.05
Miscarriage rate and Age	1.007	(0.973, 1.042)	> 0.05

OR, odds ratio; CI, confidence interval.

## Discussion

Our study revealed that in patients with simple TFI who chose frozen-thawed embryo transfer, age is an independent risk factor significantly affecting the clinical pregnancy, live birth, and miscarriage rates. In the general cohort without limiting the age of oocyte retrieval, the live birth rate drops rapidly after the female embryo transferred age exceeds 34 years, whereas the miscarriage rate increases quickly after 35 years old. However, as the age of embryo transfer increases, the clinical pregnancy, live birth, and miscarriage rates do not change significantly in women ≤ 34 years of age when oocytes are taken. These results indicate that it is the aging of oocytes rather than the aging of the body that has a significantly greater impact on the clinical pregnancy outcome of women with simple TFI.

The determinants of female fertility include gametes, fertilization, uterine receptivity, and embryo-uterine cross-talk^10^. Decrease in oocyte quality is one of the main factors that worsen pregnancy outcomes with age increase^11^. Female ovaries have a limited number of germ cells^12^, and the number of oocytes is determined by genetics^13^. The number of oocytes is gradually reduced with age increase^14,15^ until the gamete pool is completely depleted^12^, marking the arrival of female menopause and the end of childbearing age^13^. Studies^16,17^ have found that the number of follicles and the inhibin B levels produced by granular cells decrease with age. Inhibin B can inhibits secretion of FSH in the hypothalamus-pituitary^17^, and the FSH level in the early follicular phase increases as the level of inhibin B decreases, which causes more primordial follicles to be consumed in the follicle recruitment progress^18^.

DNA labeling of oocytes carried out in the early and late stages of fetal ovum development proves that the first germ cell undergoing meiosis at ovum formation of the fetus ovulates first in adults, and the last oocyte undergoing meiosis ovulates finally in adults^19^. This means that oocytes ovulating from older women have experienced more cycles of cell division at ovum formation of the fetus and received more active oxygen exposure than younger women^20^. The telomere hypothesis has a very important position in this field. Telomeres are repeating sequences of DNA that protect the ends of chromosomes and promote homologous chromosome pairing and formation in the early stages of meiosis. Telomeres become shortened and depleted as cells divide. David et al^21^ have found that shortened telomeres cause apoptosis in human preimplantation embryos. Age-related cell meiosis dysfunction is also related to shortening of telomeres^22^.

In addition, unlike sperm, oocytes carry cytoplasm from the mother, including organelles such as mitochondria and spindle. The mitochondrial genome is extremely susceptible to damage by reactive oxygen species and genotoxicity, which is related to aging damage in long-lived, post mitotic cells^23^. During the process of cell meiosis, the spindle is responsible for separating homologous chromosomes during meiosis I and sister chromatids during meiosis II to produce haploid oocytes. Damage to the spindle leads to obstacles to chromosome separation in oocytes and an increase in aneuploid gametes^24^. The probability of spindles distortion and microtubule system disorder during meiosis increases with increase of the maternal age^25^. It has been found that in women over 40 years old, nearly 80% of oocytes have abnormal spindle structure or chromosome arrangement, while in women under 25 years old, less than 20% of oocytes have abnormal spindles^26^. This may indicate that age-related spindle and chromosomal abnormalities may cause a decline in female fertility. In addition, older women's oocytes have been observed to have thicker zona pellucida and more irregular shapes^27^, which may result in female fertilization decrease but an increase in the abnormal fertilization rate^28^. In older female oocytes, the probability of chromosomal nondisjunction increases^29,30^, and the fragility of chromosomes^28^ may increase to damage the oocyte. The relationship between hypoxic environment and poor oocyte quality has been observed^31^, suggesting that blood vessels in the follicle microenvironment are damaged with age to cause hypoxia of the follicles^32^, which may further damage the oocyte. These results indicate that oocytes are the main targets of age increase and play an important role in the decline of fertility in aged women.

Our study is the first to quantify the relationship between the female age of embryo transfer and the clinical pregnancy outcome in patients with simple TFI who undergo frozen-thawed embryo transfer. The limitations of this study included retrospective design, Chinese women enrolled only, and one single center study. The specific causes of tubal infertility in these patients were not collected, which may be one limitation of the study. Although strict inclusion and exclusion criteria had been adopted and bias adjusted in this study, the professionalism and experience of the clinician for diagnosing simple TFI which is an exclusionary diagnosis may affect the accuracy of diagnosis. In the future, prospective large-scale clinical trials with multiple centers involved and multiple ethnicities enrolled are still needed to confirm the results of this study.

In summary, in patients with simple TFI undergoing IVF/ICSI frozen-thawed embryo transplantation, age is an independent risk factor significantly affecting the clinical pregnancy, live birth, and miscarriage rates. In the whole cohort without restriction of the age of oocyte retrieval, the clinical pregnancy and live birth rates decrease while the miscarriage rate increases with increase of age for embryo transfer. However, for oocyte retrieval in women ≤ 34 years of age, the clinical pregnancy, live birth, and miscarriage rates do not have significant change with the increase of embryo transfer age. It is the aging of oocytes rather than the aging of the body that has a greater impact on the clinical pregnancy outcome in women with simple TFI. Patients with pure TFI can freeze embryos in advance to preserve fertility.

## Materials and methods

### Subjects

This study was approved by the ethics committee of the Second Hospital of Hebei Medical University, and all patients had given their signed informed consent to participate. All methods were performed in accordance with the relevant guidelines and regulations. Simple tubal factor infertility refers to a type of infertility in which women cannot be fertile simply due to blockage, adhesion, and patency of the fallopian tubes after excluding other causes of infertility. It was confirmed by laparoscopically or hysterosalpingography (HSG). Patients with simple TFI who underwent IVF/ICSI frozen-thawed embryo transfer at our hospital from January, 2012 to April, 2019 were retrospectively enrolled. The inclusion criteria were patients with simple TFI who had the chromosome of 46 (XX), frozen-thawed embryos transfer, and hormone replacement protocol for preparing the endometrium. The exclusion criteria were chromosomal abnormality in the husband and/or wife, uterine or endometrial factors affecting pregnancy including uterine malformations and fibroids, adenomyoma, endometrial polyps, intrauterine adhesions, endometrial tuberculosis history, and presence of hydrosalpinx which returned to the uterine cavity. Patients who had immune diseases like systemic lupus erythematosus and Sjogren's syndrome and who were having glucocorticoid drugs and immunosuppressive agents (such as hydroxychloroquine and cyclosporine) were also excluded. A total of 3619 patients who met the inclusion criteria were enrolled and divided into the younger group (≤ 32 years of age, n = 2705) and older group (> 32 years of age, n = 914) according to the age of the patients.

### Treatment plan

All patients adopted the Gonadotropin releasing hormone agonist (GnRH-a) long protocol, that is, daily injection of triptorelin acetate (Triptorelin, Ferring GmbH, specifications: 1 ml; 0.1 mg) 0.03 mg or 0.05 mg was started in the middle of the luteal phase of the previous menstrual cycle. The serum hormone levels and B-ultrasound results were checked on the 3rd to 5th day of the menstruation cycle, and then, gonadotropin (Recombinant Human Follitropin Alfa for Injection, MerckSeronoS.p.A, Geneva, Switzerland, Specification: 5.5 μg (75 IU)) was used after the down-regulation standard had been reached (follicle-stimulating hormone (FSH) ≤ 5 IU/L, luteinizing hormone (LH) ≤ 5 IU/L, estradiol (E2) ≤ 50 pg/mL,Endometrial thickness ≤ 5 mm). The initial dose of gonadotropin was 125-375 IU per day, which was depended on the patient's age, body mass index (BMI), basal follicle number, basal serum follicle-stimulating hormone (bFSH), and baseline anti-Mullerian hormone (bAMH) level. The dose of gonadotropin was later adjusted according to the size of the follicle and the hormone results.

Chorionic Gonadotrophin for Injection (Livzon Pharmaceutical Group Inc., Zhuhai, China, specification: 2000 IU) in the dose of 6000-12000 IU was injected when the diameter of the largest follicle was bigger than 18 mm or the diameter of at least 3 follicles was bigger than 17 mm. The dose was determined according to the BMI of the patient and the serum Estradiol (E2) level on the trigger day.

Thirty-six to 37 h after injection of human chorionic gonadotropin (HCG), the oocytes were retrieved under ultrasound guidance. After the oocytes were collected, a short-term fertilization scheme was used, and the fertilization was judged by observing the formation of the second polar body. The cleavage was observed 48 h after taking the oocyte, and 72 h later, the embryos of grade I, II, and III were selected as transplantable embryos after being frozen based on the embryo rating criteria. Class I embryos were defined as those with regular blastomere morphology, uniform size, translucent, no cytoplasm, complete zona pellucida, and fragmentation rate of 0–5%. Class II embryos were those with slightly irregular blastomere morphology, slightly uneven size, granular cytoplasm, and fragmentation rate of 6–20%. Grade III embryos were those with irregular blastomere morphology, obviously uneven size, obviously granular cytoplasm, and fragmentation rate of 21–50%.

Hormone replacement protocol was used to transform the endometrium of the patient, with oral administration of progynova (Delpharm Lille SAS, Bayer Leverkusen, Germany, specification:1 mg), 2–3 mg twice daily started on the 3rd day of menstruation. The patients returned to the hospital from the 12th day of menstruation, and progesterone 40–60 mg was administered intramuscularly for luteal support for patients with the endometrial thickness ≥ 8 mm and E2 ≥ 200 pg/ml. On the fourth day of corpus luteum support, frozen-thawed embryos were transplanted to the patient. All transferred embryos were cleavage stage embryos. The frozen embryos were thawed according to the rapid recovery method of vitrification, and the surviving blastomeres with thawing > 50% was used for transplantation.

### Definition of results and indicators

The following data of demography and pregnancy outcomes were analyzed including female age, infertility duration, number of pregnancies, BMI, basial gonadal hormone concentrations, endometrial thickness on the trigger day, number of embryos transferred, clinical pregnancy rate, live birth rate, miscarriage rate, and ectopic pregnancy rate. Biochemical pregnancy was defined as serum HCG higher than 25 U/L in 12–14 days after transfer. Gynecological ultrasound examination was performed 30–40 days after transfer, and the appearance of gestational sac was considered clinical pregnancy, with the clinical pregnancy rate = (number of clinical pregnancy cycles / number of all transfer cycles) × 100%, and live birth rate = (number of live birth cycles / number of all transfer cycles) × 100%. If the pregnancy was less than 28 weeks and the fetus weighed less than 1000 g when the pregnancy was terminated, it was defined as abortion, with the abortion rate = (number of abortion cycles / number of all clinical pregnancy cycles) × 100%. Ectopic pregnancy was defined as the implantation and development of a fertilized egg outside the uterine cavity, with the ectopic pregnancy rate = (number of ectopic pregnancy cycles / number of all clinical pregnancy cycles) × 100%.

### Statistical analysis

The statistical analysis was performed with the statistical packages R (The R Foundation; version 3.4.3) and Empower (R) (X&Y solutions, Boston, MA, USA). Data with the normal distribution were expressed as mean ± standard deviation (SD), and data with the non-normal distribution were expressed as median ± quartile range (QR). For data with the normal distribution, two independent samples test was used to compare the means between the two groups, and one-way ANOVA was used to compare means among multiple groups. For data with the non-normal distribution, the non-parametric test (Mann–Whitney U-test) was used to compare the means. The chi-square test or Fisher exact probability method was used for comparison of counted data. Univariate and multivariate logistic regression analyses were performed to analyze various factors affecting clinical outcomes before and after adjustment of the confounding factors. After adjusting confounding factors, the smooth curve fitting was used to observe the relationship between transplantation age and pregnancy outcome. The smooth curve fitting and threshold effect value were combined to quantify the effect of female age on clinical pregnancy outcomes with or without restriction on the age of oocyte retrieval. The statistically significant *P* was set at < 0.05.
